# How do digital lives affect resident mental health in the digital era? Empirical evidence based on Chinese general social survey

**DOI:** 10.3389/fpubh.2022.1085256

**Published:** 2022-12-07

**Authors:** Yan Chen, Mengyang Wei, Jaime Ortiz

**Affiliations:** ^1^School of Economics and Management, Beijing University of Posts and Telecommunications, Beijing, China; ^2^Robert C. Vackar College of Business and Entrepreneurship, The University of Texas Rio Grande Valley, Edinburg, TX, United States

**Keywords:** digital lives, mental health, mediating mechanism, personal perception, social perception

## Abstract

Having good mental health means we are better able to connect, function, cope and thrive. The widespread application of digital technology in daily life provides new ways and promising tools for residents to maintain their mental health. Given the importance of mental health for everyone, and the fact that mental health problems are prevalent worldwide, this study discusses how digital lives affects the mental health of residents. The results suggest that digital lives are significantly and positively associated with mental health. Mechanisms analysis identifies personal perceptions (self-rated physical exercise and subjective wellbeing) as the important paths for digital lives to promote mental health, while social perceptions (social trust and social fairness) play a suppressing effect on the relationship between them. The results of further discussion show that the degree of the influence of digital lives on mental health of individuals is heterogeneous among different regions. Due to the difference in development level, the positive impact of digital lives is greater in urban areas than in rural areas, and it is stronger in western regions than in eastern and central regions. This study enriches the nascent research stream of digitalization, explores new paths of harnessing digital technologies for mental health, and offers useful insights for the government to guide them in formulating digital development strategies and achieving the Healthy China Strategy.

## 1. Introduction

Mental health is believed to be essential to a happy, satisfying, and meaningful life. It is “an integral part of our general health and wellbeing and a basic human right” (1). The reality, however, is that nearly one billion people worldwide suffered from mental disorders in 2019, and that mental disorders (such as depression and anxiety) are among the top 10 causes of the global burden of disease (2). In addition, the COVID-19 pandemic has taken huge toll on people's mental health. According to the World Health Organization, rates of depression and anxiety went up by more than 25% in the first year of the pandemic.

Beyond mental health itself, the far-reaching effects of digitalization on mental health cannot be ignored (3). Digitalization in our living environment is augmented by the continuous innovation and integrated application of the underlying digital technology. For example, the use of digital media (such as computers, mobile phones, video websites, and social media) has become quite common and has become a new way to access mental health information and support (4). Lockdown and restrictions in movement and social contact due to the COVID-19 pandemic have led to increased reliance on a digital lifestyle, such as accessing health care services (5, 6). While digitalization has a variety of favorable functions, excessive usage of a kind of digital product, such as a smartphone, has the paradoxical effect of diminishing the mental health of its users (7). The relationship between psychological symptoms and the use of Internet and new media is likely to be even more complex than existing study was able to elucidate (8). As such, it is essential to explore the potential impact of digital lives on the overall mental health of people in greater depth.

The previous literature on the relationship between digital lives and mental health was inconclusive, three seemingly arguments exist. The first opinion is that digitalization will promote mental health, as people who use the Internet more frequently have substantially lower odds of having mental health problems (9). The second is that overuse of universal digital technologies or digital products, particularly in the form of addiction to the Internet, will cause disturbances or harm to the individual's mental health (10, 11). The third view holds that there are some indirect impact mechanisms between digitalization and mental health. One is the interpersonal emotion explanation mechanism which holds that aspects of digital lives, including online social activities, leisure, and entertainment, can increase subjective wellbeing and reduce stress and depression levels (12–14). Another is the information acquisition explanation mechanism which holds that many websites can be available to seek mental health information and support online (15, 16).

A literature review reveals that scholars have attempted to understand how digitalization of life is shaping mental health. Nevertheless, the relationship between digital lives and mental health remains unclear. In the further exploration of the influencing mechanism, the existing research mainly takes two approaches: the interpersonal emotion explanation mechanism and the information acquisition explanation mechanism. As individuals in a complex social environment, people's psychological health and wellbeing are related to their social environment to some extent (17). However, the literature on interpersonal emotion has generally focused only on a single dimension—emotion perception at the individual level—ignoring the emotional perception at the social level. In addition, some studies suggest that only a minority of digital users take advantage of digital technology for mental health purposes (18, 19). The Internet seems to be underutilized for information on mental health, and its usage is primarily focused on entertainment, watching media, listening to music, playing games, social communication, and similar activities. Therefore, information access mechanisms do not seem to explain the relationship between digitalization of life and mental health.

Accordingly, our study proposes the following two research questions (RQs):

RQ1: What is the impact of digital lives on mental health?
RQ2: Through what emotion perception mechanism do digital lives influence mental health at multi-dimensions?

To answer these questions, we developed a theoretical model and validated it using data from the Chinese General Social Survey (CGSS), which was carried on the Department of Social Sciences, Renmin University of China in cooperation with the Survey Research Center of the Hong Kong University of Science and Technology. This study makes three theoretical contributions to the literature. First, this paper provides new empirical evidence to further understand digitalization and its impact on mental health, which enriches the research on digital lives and mental health. Second, this paper attempts to clarify the mechanism of the impact of digital lives on mental health from multiple dimensions, including individual perception dimension and social perception dimension, which fill the theoretical gaps in mental health research on digitalization. Third, based on existing research, this paper compares different groups of residents to further clarify the relationship between digital lives and mental health in order to provide a realistic basis for better guidance on using digitalization to enhance the mental health of residents. In addition, this paper has important practical significance. First, this paper offers useful insights for the government and guides the government in formulating digital development strategies and achieving the Healthy China Strategy. Second, this paper provides specific coping methods for residents to make full use of digitalization to maintain their mental health. Third, this paper analyzes the possible shortcomings of social ethics in the context of digital transformation and provides useful ideas for the government to provide mental health intervention measures and related system construction.

The rest of the paper is structured as follows: Section 2 provides theoretical underpinning based on the related literature and presents the hypothesis. Section 3 describes methodology of this study, including data sources, variables selection and measurements. Section 4 presents model selection and the main results. Section 5 provides further discussion based on the results of the full sample and sub-sample, respectively. Section 6 summarizes the research conclusions.

## 2. Theoretical underpinning and hypothesis development

With digital technology increasingly integrated into our daily lives, digitalization has become a trend, as most daily activities have now gone online (20, 21). In the other words, people can enjoy shopping online, adopt digital payment, work from home, and communicate with others at will without leaving home (22–25). The status of mental health can also be affected by the convenience of digitalization. Although some studies suggest that digitalization could harm the mental wellbeing of the individual (26, 27), this negative digitalization usually refers specifically to problematic Internet use (PIU), which is defined as the excessive use of the Internet, which causes disturbances or harm to the individual (28), for example, through online gaming and cyber pornography. In general, the emerging of digital lives has been contributing to improving the overall condition of mental health (29), which is mainly reflected in in the following two aspects. First, some medical interventions can be delivered *via* digital technologies (30). Due their relatively low cost and ease of scalability, digital health interventions such as apps, digital platforms, and wearables, will help bring access to mental health support (31). Second, there are many benefits of interacting with others through the Internet use. Online communication has become a pathway for family members to maintain relations and share affection (26), and it has become an important way for individuals to receive peer support and to connect with others with similar experiences (32). In sum, digital lives enable individuals to access external mental health support, maintain family ties, and increase interpersonal communication, thereby allowing them to relax and release stress. Following the above analysis, we propose H1:

- H1: Digital lives are positively correlated with the mental health of residents.

With the development of digitalization in people's daily life, such as the application of smartphones, wearables, and sensors, more and more fitness apps have emerged in the market to provide users with personalized, scientifically reasonable fitness plans, meet users' various fitness needs and guide users to adjust according to their actual physical conditions (33, 34). According to recent statistics, the Health and Fitness category accounts for a large proportion of apps in both the Android and Apple app stores—the eighth-largest categories of apps (35). Previous literature has shown that smartphone app usage is one of the most frequently used methods of digital health interventions for enhancing physical activity (36), and the use of apps can increase cognitive patterns encouraging exercise and physical activity, so those who use fitness apps participate in significantly more physical activity than those who do not (37). A growing literature recognizes the positive effects of exercise on emotional states such as anxiety, stress and depression, including helping people with mood disorders achieve better mental health outcomes (38). In actual treatment, mental health practitioners view exercise as an effective evidenced-based intervention for a range of mental health conditions, and they often prescribe exercise regularly to patients who are experiencing anxiety, stress, and depression (39). Various mechanisms have been proposed to explain the positive effect of physical activity on mental health. For instance, exercise may reduce stress hormones and increase the levels of endorphins and brain-derived neurotrophic factor in the body, which could make people feel happy, optimistic, and relaxed. Exercise can also help distract people from stress and improve their ability to control overly stressful situations (40). Following the above analysis, we propose H2a:

- H2a: Digital lives improve mental health of residents by enhancing physical exercise.

Previous studies have found that digitalization of life is closely related to subjective wellbeing, that the use of digital technology can affect wellbeing in different ways, and that it can positively predict subjective wellbeing (41, 42). For example, as a device to support independent living and social activities, the smartphone increases the opportunities to contact with the outside world and enables people to have more social connections and support. In addition to providing users with always-on connectivity, the widespread use of smartphones in daily life affords users access to burgeoning information and a variety of entertainment options. The social use, informational use, and entertainment use of smartphones will greatly enhance people's wellbeing and lead to a more positive attitude toward life (43). Digital lives' contribution to subjective wellbeing has proved to be age-neutral. Children can get playful consumption experience, enjoyment, and sensory experiences (44), and adolescents and older adults can alleviate feelings of loneliness and isolation through online communication (45, 46). Subjective wellbeing at the psychological level, as described above, is often identified as a state of positive mental health. Some studies of the relationship between subjective wellbeing and negative spiritual conditions, such as anxiety and depression, show that there is a significantly inverse correlation between subjective wellbeing and depression, death anxiety, pain interference and other negative psychological conditions (47, 48). Accordingly, the stronger the subjective wellbeing, the better the mental health. In sum, subjective wellbeing is a powerful predictor of mental health, even better than positive changes in outlook (49). In other words, wellbeing can effectively predict mental health. Following the above analysis, we propose H2b:

- H2b: Digital lives improve the mental health of residents by improving subjective wellbeing.

One controversial phenomenon in the digital era is that, while digitalization increases information transparency and improves communication channels, which provides an opportunity to recovery or re-build trust, digitalization is also leading to the decline of trust. There are two main explanations for this. First, the negative effects of information overload on social trust should not be ignored (50). The digitalization of all aspects of life has created a huge amount of information, but due to the existence of information inequality and lack of effective information filtering, individuals in the digital world are easily exposed to misleading news, fraud, and various social scandals (51). Second, incongruency between advertisement shared on social media and the online motivation may increase privacy concerns, which in turn fuel social distrust (52). Social trust levels in the digital context may further influence mental health, current research suggests that the mental health of residents who trust and help each other is significantly higher than that of residents without trust and mutual help (53). In other words, the low level of interpersonal trust in society is one of the important risk factors for mental health (54). A growing body of evidence suggests that interpersonal trust might be associated with mental health outcomes; for example, trust is negatively related to mental illness, and people who have high interpersonal trust are less likely to experience suicidal ideation than those who have low interpersonal trust (55, 56). Therefore, social trust may suppress the positive association between Internet use and mental health. Following the above analysis, we propose H2c:

- H2c: Digital lives endanger the mental health of residents by reducing their sense of social trust.

Inequalities in the distribution of resources and opportunities persist around the world. The advancement of digital technology and digitalization of life will play a significant impact on an individual's perceptions toward these aspects of social fairness. There is evidence that Internet use negatively affects social fairness perceptions directly by channeling their social emotions (57). While information can be easily accessed online, negative news, including the unequal distribution of educational resources, excessive income, large urban-rural gaps, and political corruption, is also widely disseminated through Internet, which may change individual's perceptions of the society where they live, especially the level of social fairness (58). Some people who suffered injustice in real life try to seek justice online by uploading their experience to social media, and the justice-seeking posts will quickly be viewed and shared by thousands of social media users (59), and thus sympathy toward the unfairly treated groups arouses empathy and negative judgment on social fairness. Belief in social fairness has salutary effects for mental health from a wide range of individuals (60); whereas inequality has its most fundamental effects on the prevalence of number of psychopathologies (61) and a statistically significant positive relationship between inequality and risk of depression has been reported (62). Due to the Internet widening the scope of social comparisons, it can generate a sense of relative deprivation and frustration that negatively affects mental health. With the perception of social fairness fades, psychological deficits, including a poor sense of self, unmet needs, and personal trauma, will be generated in the public, which may even trigger hatred in irrational scenarios (63). Following the above analysis, we propose H2d:

- H2d: Digital lives endanger the mental health of residents by weakening a sense of social fairness.

**Figure 1:**
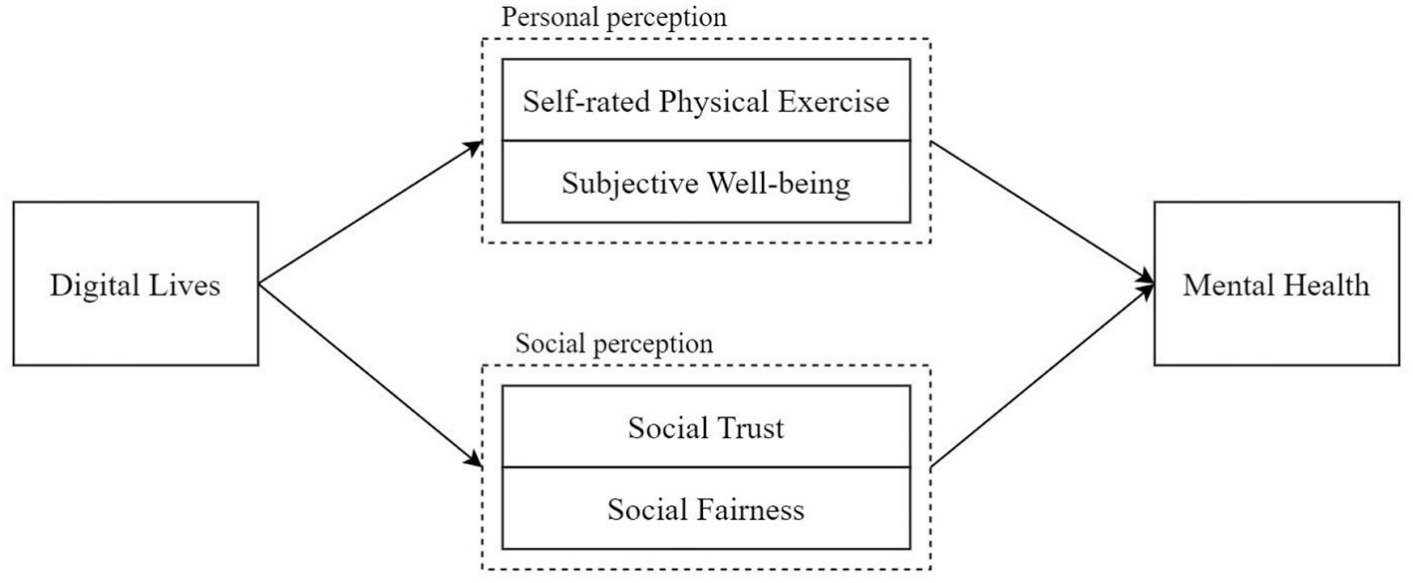
Research framework.

Based on the above research hypotheses, we construct the research framework, as shown in Figure 1.

## 3. Data sources and specification of variables

### 3.1. Data sources

The data used in this paper are mainly from the Chinese General Social Survey (CGSS). Specifically, CGSS is a national, comprehensive academic survey that collects data at multiple levels of society, community, family, and individuals. CGSS data are widely used in studies of health and Internet use (64–67). Considering the timeliness and availability of the data, we selected cross-sectional data from the latest released 2017 CGSS as the samples for this study. Independent variables, dependent variables, mediating variables and control variables at the individual and family levels are all derived from the above survey. In addition, part of the control variables at the regional level is collected from the Institute of Digital Finance Peking University. We finally retained 12,085 observations after deleting missing values and outliers and eliminating the data that answered “Not applicable,” “Refused to answer,” and “Don't know.”

### 3.2. Variables selection

#### 3.2.1. Dependent variable

The dependent variable is mental health of residents, measured by responses to the question, “How often did you feel depressed in the past four weeks?”. We assign 1 to “Always,” 2 to “Often,” 3 to “Sometimes,” 4 to “Rarely,” and 5 to “Never.” The better the mental health, the higher the value.

#### 3.2.2. Independent variable

The independent variable is digital lives, measured by responses to the question, “How often did you use the Internet (including mobile Internet access) in the past year?”. We assign 1 to “Never,” 2 to “Rarely,” 3 to “Sometimes,” 4 to “Often,” and 5 to “Always.” The more frequently the Internet is used (that is, the higher the value), the higher the degree of digitalization of lives.

#### 3.2.3. Mediating variables

The mediating variables in this study are set from two dimensions: personal perception and social perception. Specifically, the mediating variables of the individual perception dimension are self-rated physical exercise and subjective wellbeing. Self-rated physical exercise is measured by responses to the question, “In the past 12 months, how many times per week did you typically engage in 30 min of physical activity that made you sweat?”; and subjective wellbeing is measured by responses to the question, “In general, do you feel your life is happy?” The mediating variables of the social perception dimension are social trust and social fairness. We use the answer to the question, “In general, do you agree that the majority of people in society can be trusted?” as the proxy variable for social trust and the answer to the question, “In general, do you think that today's society is fair?” as a proxy variable for social fairness. Except for self-rated physical exercise is expressed by the frequency of physical exercise filled in by respondents, the values of the other three mediating variables are divided into five levels, ranging from 1 to 5, with the higher values indicating greater senses of happiness, social trust, and justice.

#### 3.2.4. Control variables

The control variables include the individual, family, and regional control variables. We select age, gender, household registration, education level, marital status, and health insurance of respondents as control variables at the individual level, select Internet access status of family members as control variables at the family level, and select the regional digital financial inclusion index as control variables at the regional levels.

The specific definitions and assignments of the variables are shown in Table 1.

**Table 1:** Variable definition and assignment.

**Variable type**	**Variable code**	**Variable assignment**
Dependent variable	MH	From 1 to 5, the better the mental health, the higher the value
Independent variable	DL	From 1 to 5, the higher the degree of digitalization of lives, the higher the value
Mediating variables	Exercise	Frequency of physical exercise at least 30 min
	Happiness	From 1 to 5, greater sense of happiness, the higher the value
	Trust	From 1 to 5, greater sense of social trust, the higher the value
	Fairness	From 1 to 5, greater sense of social fairness, the higher the value
Control variables	Age	Survey year minus birth year
	Gender	Male = 1, Female = 0
	Household	Agricultural household registration = 1, Non-agricultural household registration = 0
	Education	From 1 to 13, the higher the level of education, the higher the value
	Married	Married and living with their spouse = 1, Other = 0
	Insurance	Yes = 1, No = 0
	Family	Residents with other family members surfing the Internet = 1, Residents without other family members surfing the Internet = 0
	DFII	Regional digital financial inclusion index

### 3.3. Descriptive statistics

Descriptive statistics and correlations of the variables are shown in Table 2. The average mental health is 3.804, which indicates that resident mental health status is fair but still requires improvement. There is a significant positive correlation between the core variables MH and DL, which preliminarily supports the basic hypothesis of this paper that digital lives have a positive impact on the mental health of residents. In addition, in the process of regression analysis, we have conducted a variance inflation factor test. The results show that the overall VIF mean is 1.51 and the VIF coefficient of each independent variable do not exceed 10, which further indicates that there is no multicollinearity problem in this paper.

**Table 2:** Descriptive statistics and correlation coefficient matrix of variables.

	**(1)**	**(2)**	**(3)**	**(4)**	**(5)**	**(6)**	**(7)**	**(8)**	**(9)**	**(10)**	**(11)**	**(12)**	**(13)**	**(14)**
	**MH**	**DL**	**Exercise**	**Happiness**	**Trust**	**Fairness**	**Age**	**Gender**	**Household**	**Education**	**Married**	**Insurance**	**Family**	**DFII**
(1)	1													
(2)	0.158***	1												
(3)	0.130***	0.122***	1											
(4)	0.316***	0.093***	0.118***	1										
(5)	0.082***	−0.100***	0.019**	0.174***	1									
(6)	0.125***	−0.096***	0.016*	0.296***	0.308***	1								
(7)	−0.060***	−0.640***	0.023**	0.017*	0.136***	0.122***	1							
(8)	0.062***	0.036***	0.039***	−0.034***	0.00400	0.020**	0.0100	1						
(9)	−0.157***	−0.283***	−0.212***	−0.118***	0.0100	−0.00300	−0.027***	−0.00700	1					
(10)	0.168***	0.594***	0.148***	0.134***	−0.019**	−0.0110	−0.461***	0.096***	−0.454***	1				
(11)	0.044***	−0.045***	−0.010	0.062***	0.020**	−0.025***	0.085***	0.0100	0.047***	−0.121***	1			
(12)	0.010	0.000	0.017*	0.051***	0.033***	0.028***	0.044***	0.00300	−0.018*	0.035***	0.066***	1		
(13)	0.101***	0.433***	0.084***	0.103***	−0.049***	−0.061***	−0.329***	−0.041***	−0.161***	0.288***	0.032***	0.027***	1	
(14)	0.169***	0.225***	0.139***	0.096***	−0.016*	−0.034***	0.030***	−0.00600	−0.369***	0.262***	−0.034***	0.0130	0.136***	1
Mean	3.804	2.831	2.085	3.864	3.465	3.1	50.769	0.472	0.538	5.195	0.78	0.924	0.774	282.154
Std. Dev.	0.996	1.721	3.035	0.848	1.031	1.064	16.658	0.499	0.499	3.275	0.415	0.266	0.418	27.679

***p < 0.01, **p < 0.05, *p < 0.1.

## 4. Model selection and empirical results

### 4.1. Model selection

To explore the impact of digital lives on mental health, we first used the OLS model to perform a preliminary regression. We then constructed the mediating effect model by adding the mediating variables (Exercise, Happiness, Trust, and Fairness), referring to the mediation effect test procedure proposed by Wen and Ye (68), to test whether digital lives affect mental health through self-rated physical exercise, subjective wellbeing, social trust, and social fairness. The models are set as follows:


(1) $$\begin{array}{lll} MH_{i} & = & {\alpha_{1}+\beta_{1}DL}_{i}+\gamma_{1}CV_{i}+\varepsilon_{i} \end{array}$$

(2) $$\begin{array}{lll} M_{i} & = & {\alpha_{2}+\beta_{2}DL}_{i}+\gamma_{2}CV_{i}+\varepsilon_{i} \end{array}$$

(3) $$\begin{array}{lll} MH_{i} & = & {\alpha_{3}+\beta_{3}DL}_{i}+\delta M_{i}+\gamma_{3}CV_{i}+\varepsilon_{i} \end{array}$$

Where *MH*_*i*_ represents the mental health status of residents, *DL*_*i*_ represents the degree of digitalization of lives, *CV*_*i*_ represents other factors that affect mental health, and ε_*i*_ represents the random disturbance term. β_1_ is the coefficient we focus on, reflecting the total direction and extent of the impact of digital lives on mental health. In the mediating effect test, *M*_*i*_ represents the mediating variables. The testing procedures are as follows: Step 1 is to test the coefficient β_1_ in Equation (1), and if it is significant, the mediating effect is established, and the follow-up inspections are carried out. Step 2 successively tests β_2_ in Equation (2) and δ in Equation (3), and if they are significant, it means that the indirect effect is significant, and then step 4 is carried out; if at least one is not significant, perform step 3 at a later test. Step 3 is to use the Bootstrap method to test the null hypothesis: β_2_×δ = 0, and if it is significant, it indicates that the indirect effect is significant, and then step 4 is carried out. Otherwise, the analysis stops. Step 4 is to test the coefficient β_3_ in Equation (3), and if it is not significant, the direct effect is not significant, indicating that the model only has a mediating effect; if it is significant, go to step 5. Step 5 is to compare the signs of β_2_×δ and β_3_, and if the signs are consistent, it means that a partial mediating effect exists, and if the signs are different, it means that a suppressing effect exists.

### 4.2. Empirical results

The results of the baseline regression are shown in Table 3. In Models (1) and (2), the coefficients of digital lives (DL) are all significantly positive (*p* < 0.01), indicating that the digitalization of lives can effectively improve the mental health of residents. Thus, H1 is supported. Concerning the control variables in Model (2), the results indicate that higher levels of education and development of regional digital financial inclusion are helpful in improving residents' mental health. Compared with women, agricultural household registration residents, unmarried people, and residents without other family members using the Internet, men, non-agricultural household registration residents, married people, and residents who have family members using the Internet have advantages in the impact of digital lives on mental health.

**Table 3:** Baseline regression results.

**Variable**	**(1) MH**	**(2) MH**
DL	0.0917***	0.0442***
	(0.0052)	(0.0080)
Age		0.0012
		(0.0008)
Gender		0.1072***
		(0.0178)
Household		−0.1226***
		(0.0217)
Education		0.0209***
		(0.0037)
Married		0.1416***
		(0.0217)
Insurance		−0.0018
		(0.0343)
Family		0.0718***
		(0.0244)
DFII		0.0039***
		(0.0004)
Constant	3.5441***	2.2552***
	(0.0179)	(0.1162)
Observations	12,085	12,085
R-squared	0.0251	0.0594

Robust standard errors in parentheses, ^***^p < 0.01.

### 4.3. Robustness test

In this study, we adopt two methods to test the robustness of the baseline regression results. The first method is to use the ordered logit model for regression, considering that mental health is typical ordinal data. The second method is to replace the original independent variable by whether to use Alipay or WeChat payment, because the adoption of electronic payment means can also reflect the degree of digitalization of a person's life to a certain extent. In Table 4, Model (1) is the Ologit estimation results of the impact of digital lives on mental health. Model (2) and Model (3) are the OLS estimation results under the substitution variable method, respectively. Among them, Digital1 represents whether WeChat payment has been used. If the answer is yes, it is 1; otherwise, it is 0. Digital2 represents whether Alipay has been used. If the answer is yes, it is 1; otherwise, it is 0. It can be seen from Table 4 that the coefficients of independent variables in three models are all significantly positive, indicating that the estimated result is still robust.

**Table 4:** Robustness test.

**Variable**	**(1)**	**(2)**	**(3)**
	**MH**	**MH**	**MH**
DL	0.0825***		
	(0.0153)		
Digital1		0.0914***	
		(0.0268)	
Digital2			0.0670**
			(0.0264)
Age	0.0031**	0.0005	−0.0001
	(0.0015)	(0.0008)	(0.0008)
Gender	0.2142***	0.1093***	0.1099***
	(0.0337)	(0.0178)	(0.0178)
Household	−0.2242***	−0.1361***	−0.1398***
	(0.0406)	(0.0216)	(0.0215)
Education	0.0402***	0.0233***	0.0237***
	(0.0071)	(0.0037)	(0.0037)
Married	0.2807***	0.1478***	0.1502***
	(0.0406)	(0.0217)	(0.0218)
Insurance	−0.0229	−0.0058	−0.0053
	(0.0657)	(0.0344)	(0.0344)
Family	0.1262***	0.0902***	0.0965***
	(0.0451)	(0.0240)	(0.0238)
DFII	0.0088***	0.0041***	0.0041***
	(0.0007)	(0.0004)	(0.0004)
/cut1	−0.8173***		
	(0.2357)		
/cut2	1.0883***		
	(0.2280)		
/cut3	2.7686***		
	(0.2287)		
/cut4	4.3362***		
	(0.2317)		
Constant		2.3153***	2.3471***
		(0.1162)	(0.1153)
Observations	12,085	12,036	12,036
R-squared/Pseudo R2	0.0243	0.0578	0.0574

Robust standard errors in parentheses, ^***^p < 0.01, ^**^p < 0.05.

### 4.4. Mediating effects test

Table 5 reports the mediating effects of self-rated physical exercise, subjective wellbeing, social trust, and social fairness. The test result of step 1 in column (1) of Table 5 shows that the coefficient of DL is positive and significant at the 1% level, which means that there is a mediating effect on the impact of digital lives on residents' mental health. In the sequential test of step 2, we find that the variable “DL” has a significant effect on the four mediating variables. The results from columns (2) through (5), respectively, show that the improvement of the degree of digital lives increases personal physical exercise and happiness, while exacerbating social distrust and social unfairness. At the same time, the coefficients of the four mediating variables in column (6) are still significant, indicating that the indirect effects of mediating variables are significant. Based on the results of step 2, we skip to step 4 for inspection. The coefficient of DL in column (6) is still significantly positive and lower than that in column (1) after adding the four mediating variables. The result shows that direct effects are significant and that digital lives can influence residents' mental health through self-rated physical exercise, subjective wellbeing, social trust, and social fairness. Finally, we perform step 5 to compare the signs of coefficients. The sign of the product of DL's coefficient in column (2) and Exercise's coefficient in column (6), the sign of the product of DL's coefficient in column (3) and Happiness's coefficient in column (6) are consistent with the sign of DL's coefficient in column (6), indicating that self-rated physical exercise and subjective wellbeing are considered to play a partial mediating role in the relationship between digital lives and mental health. This also means that digital lives can improve the mental health of residents by promoting physical activity and increasing wellbeing. Undergoing similar analysis, the sign of the product of DL's coefficient in column (4) and Trust's coefficient in column (6), the sign of the product of DL's coefficient in column (5) and Fairness's coefficient in column (6) are different from the sign of DL's coefficient in column (6), indicating that social trust and social fairness are considered to play a suppressing effect on the relationship between digital lives and mental health. This also means that digital lives will have a negative impact on mental health of residents by reducing the sense of social trust and social fairness. To sum up, H2a, H2b, H2c, and H2d are supported.

**Table 5:** Mediating effects test.

**Variable**	**(1)**	**(2)**	**(3)**	**(4)**	**(5)**	**(6)**
	**MH**	**Exercise**	**Happiness**	**Trust**	**Fairness**	**MH**
DL	0.0442***	0.1776***	0.0302***	−0.0274***	−0.0299***	0.0336***
	(0.0080)	(0.0252)	(0.0068)	(0.0086)	(0.0089)	(0.0077)
Exercise						0.0197***
						(0.0029)
Happiness						0.3105***
						(0.0118)
Trust						0.0316***
						(0.0093)
Fairness						0.0468***
						(0.0093)
Age	0.0012	0.0223***	0.0068***	0.0096***	0.0085***	−0.0021***
	(0.0008)	(0.0024)	(0.0007)	(0.0008)	(0.0008)	(0.0007)
Gender	0.1072***	0.1762***	−0.0821***	−0.0120	0.0237	0.1286***
	(0.0178)	(0.0543)	(0.0152)	(0.0188)	(0.0193)	(0.0170)
Household	−0.1226***	−0.7911***	−0.0272	0.0833***	0.0284	−0.1025***
	(0.0217)	(0.0683)	(0.0189)	(0.0230)	(0.0235)	(0.0207)
Education	0.0209***	0.0548***	0.0352***	0.0334***	0.0311***	0.0064*
	(0.0037)	(0.0115)	(0.0031)	(0.0040)	(0.0041)	(0.0036)
Married	0.1416***	−0.0212	0.1376***	0.0325	−0.0776***	0.1019***
	(0.0217)	(0.0652)	(0.0196)	(0.0224)	(0.0231)	(0.0205)
Insurance	−0.0018	0.0656	0.1073***	0.0873**	0.0893**	−0.0433
	(0.0343)	(0.0966)	(0.0315)	(0.0368)	(0.0369)	(0.0324)
Family	0.0718***	0.2776***	0.1428***	0.0001	−0.0357	0.0237
	(0.0244)	(0.0710)	(0.0219)	(0.0247)	(0.0259)	(0.0232)
DFII	0.0039***	0.0049***	0.0009***	−0.0009**	−0.0018***	0.0037***
	(0.0004)	(0.0011)	(0.0003)	(0.0004)	(0.0004)	(0.0003)
Constant	2.2552***	−1.1269***	2.7415***	2.9841***	3.0743***	1.1880***
	(0.1162)	(0.3623)	(0.0989)	(0.1225)	(0.1245)	(0.1172)
Observations	12,085	12,085	12,085	12,085	12,085	12,085
R-squared	0.0594	0.0620	0.0456	0.0255	0.0238	0.1470

Robust standard errors in parentheses, ^***^p < 0.01, ^**^p < 0.05, ^*^p < 0.1.

## 5. Further discussion

Across these mental health outcomes, more exposure to digital lives translates to fewer psychological symptoms, and digital lives could indirectly influence mental health from the personal perception dimension and the social perception dimension, which is consistent with some previous findings (69, 70).

The above results are only the average effect of the whole sample analysis, and the differences among different groups are not considered. Given that the degrees of digitalization of life may be various in different resources and environments, the impacts of digital lives on residents' health may be heterogeneous in different regions. We selected economic regionalization and household registration types to divide the sample into groups for further discussion. Table 6 reports the regression results of the subgroup samples. Models (1) and (2) show that digital lives have a significant positive impact on mental health in both urban and rural areas, but in terms of the coefficient values, the positive impact is greater in urban areas than in rural areas. One explanation for this result is that people living in urban areas are more prone to experiencing loneliness than those living in rural areas (69), and urban residents affected by digitalization get more emotional support through online channels, thereby improving their mental health more greatly. Similarly, Model (3) through (5) reflect that the positive impact of digital lives on mental health is stronger in the group of western regions, compared with the eastern and central regions. That may be related to differences in connectivity and mobility caused by economic conditions (9). The residents in the more developed eastern and central regions have higher income levels, and could easily connect with friends and engage in a variety of entertainment forms, even without digital channels. However, these are scarce in the economically underdeveloped western regions. Consequently, digital lives can enable residents in western regions to enjoy more positive mood, which is conducive to alleviating negative emotions and promoting mental health.

**Table 6:** The regression results of the subgroup samples.

**Variable**	**(1)**	**(2)**	**(3)**	**(4)**	**(5)**
	**MH**	**MH**	**MH**	**MH**	**MH**
	**Rural area**	**Urban area**	**Eastern region**	**Central region**	**Western region**
DL	0.0349***	0.0459***	0.0333***	0.0355**	0.0505***
	(0.0115)	(0.0113)	(0.0117)	(0.0140)	(0.0179)
Control variables	Yes	Yes	Yes	Yes	Yes
Constant	2.2679***	2.2530***	2.7661***	4.0494***	2.2911***
	(0.1946)	(0.1413)	(0.1839)	(0.4850)	(0.5956)
Observations	6,496	5,589	5,425	3,847	2,813
R-squared	0.0470	0.0339	0.0275	0.0419	0.0498

Robust standard errors in parentheses, ^***^p < 0.01, ^**^p < 0.05.

## 6. Conclusion

This study empirically examined the impact of digital lives on mental health of residents as well as the underlying mechanism based on the data of CGSS in 2017. The results indicate that the penetration of digitalization in daily life has a directly positive impact on the maintenance of mental health of residents. In addition, the analysis of the mediating effect model identifies personal perception and social perception as the important path mechanisms for digital lives to affect mental health. Self-rated physical exercise and subjective wellbeing in personal perception demission play a partial mediating role in the relationship between them; however, social trust and social fairness in social perception have a suppressing effect on the relationship between them. The results of further discussion show that the degree of influence of digital lives on the mental health of individuals is heterogeneous among different regions. Due to the difference in development levels, the positive impact of digital lives is greater in urban areas than in rural areas and is stronger in the western regions than in the eastern and central regions.

Based on the above research conclusions, we make the following suggestions. First, the government should further strengthen the construction of digital infrastructure and improve the digital penetration, and especially narrow the regional digital divide, thereby increasing the opportunities for every citizen to access to digitalization, which could fully leverage the positive effects of digital lives on mental health and achieve the strategic objective of Healthy China Strategy. Second, the government should also encourage the application of digital technology and develop the digital economy consistently, thus inspiring the positive influence of digital lives on mental health for individuals, such as accessing more mental health support, contacting to friends and family members at any time, getting more recreational and leisure activities, increasing subjective wellbeing. Third, be aware of the importance of maintaining a sound digital environment. In particularly, regulatory authorities should strengthen the control and supervision of Internet media and actively guide the correct value and behavioral norms to weaken the transmission mechanisms that digital lives have a negative impact on the perception of social trust and fairness.

This study still has several limitations, which should be made further breakthroughs in the future. First, this study examined the relationship between digital lives and mental health based on cross-sectional data, which could not capture the dynamic development of mental health very well. Future research can select continuous survey data from multiple years to explore their relationship. Second, this study was conducted with populations in China, and the generalizability of the research findings is limited by the sample size and country sources. Future research can extend our study to greater numbers of respondents from other regions and countries. Finally, due to the availability of data, this study was unable to explore the impact of digital lives on the mental health of residents during the COVID-19 pandemic and in the post-pandemic era. Future research can take the COVID-19 epidemic as an external environmental factor to explore its moderating effect on the relationship between digitalization and mental health.

## Data availability statement

The raw data supporting the conclusions of this article will be made available by the authors, without undue reservation.

## Author contributions

YC: conceptualization, methodology, and writing—original draft. MW: data collection, software, formal analysis, and writing—review and editing. JO: revised the paper. All authors listed have made a substantial, direct, and intellectual contribution to the work and approved it for publication.
